# Norisoboldine, a Natural Isoquinoline Alkaloid, Inhibits Diaphyseal Fracture Healing in Mice by Alleviating Cartilage Formation

**DOI:** 10.3390/biomedicines11072031

**Published:** 2023-07-19

**Authors:** Wenliang Yan, Meng Shen, Kainong Sun, Shiming Li, Jingyuan Miao, Jun Wang, Jiayang Xu, Pengcheng Wen, Qian Zhang

**Affiliations:** 1 College of Food Science and Engineering, Gansu Agricultural University, Lanzhou 730070, China; 2 Department of Nutrition and Health, China Agricultural University, Beijing 100193, China; 3 Food Laboratory of Zhongyuan, Luohe 462300, China

**Keywords:** norisoboldine, fracture healing, BMP2, endochondral ossification

## Abstract

Norisoboldine (NOR), the major isoquinoline alkaloid constituent of a Chinese traditional medicine Radix Linderae, has been demonstrated to inhibit osteoclast differentiation and improve arthritis. The aim of this study is to examine the effect of NOR on bone fracture healing and the underlying mechanisms correlated with bone marrow stromal cells (BMSCs) differentiation to chondrocytes. Our results showed that NOR inhibits the tibia fracture healing process by suppressing cartilage formation, which leads to less endochondral ossification, indicated by less osterix and collage I signaling at the fracture site. Moreover, NOR significantly reduced the differentiation of primary BMSCs to chondrocytes in vitro by reducing the bone morphogenetic protein 2 (BMP2) signaling. These findings imply that NOR negatively regulates the healing of the tibial midshaft fracture, which might delay the union of the fractures and should be noticed when used in other treatments.

## 1. Introduction

Bone fractures are the most common organ trauma causing disability in humans, especially because of the worldwide aging-caused osteoporosis, which results in increased bone fragility and weakness, consequently inducing accumulated fracture risk. Approximately 10% of fractures do not heal properly [1]. Fracture healing is initiated by inflammatory response, which releases various biological signals to recruit immune cells and bone marrow stromal cells (BMSCs) to form a stabilizing callus around the fractured part [2]. There are two types of ossification process in fracture healing: intramembranous ossification, which is a process of direct differentiation from BMSCs to osteoblasts without a cartilaginous template, and endochondral ossification, which involves a step of BMSCs differentiation into chondrocytes and the cartilaginous callus subsequently replaced by bony tissue [3]. Fractures are healed when the calcified callus is formed at the broken site [4]; hence, chondrocyte formation and cartilage matrix calcification are both very important for fracture healing.

Norisoboldine (NOR) is the primary isoquinoline alkaloid constituent of *Radix Linderae*, which is frequently used in traditional Chinese medicine [5]. Many kinds of isoquinoline alkaloids isolated from plants have various pharmacological activities, such as anti-inflammatory, antiviral, cytotoxic, and ROS scavenger properties [6]. Using modern technologies, NOR has been proven to attenuate sepsis-induced acute lung injury [7], suppress anti-inflammatory activity in atopic dermatitis by inhibiting the nuclear factor of activated T-cells (NFAT) [8], suppress endothelial cell migration [9], etc. In the musculoskeletal system, NOR works as an agonist of the aryl hydrocarbon receptor to alleviate osteoclast differentiation and inflammatory bone erosion in rats [5], possibly by activating mitogen-activated protein kinases (MAPKs)–nuclear factor kappa-light-chain-enhancer of activated B-cells (NFκB) pathways [10].

There are several studies focused on the function of NOR in arthritis as well. In rats with drug-induced arthritis, the oral administration of NOR regulates the balance between Th17 and regulatory T-cells in the intestinal lymph nodes [11], inhibits the pleiotropic cytokine interleukin-6 (IL-6) in fibroblast-like synoviocytes [12], and promotes the Notch1 pathway in endothelial cells to prevent synovial angiogenesis [13], displaying an anti-rheumatoid arthritis (RA) property. In mice with collagen-II-induced arthritis, NOR treatment alleviated the disease by reducing the infiltration of inflammatory cells, synovial hyperplasia, and protecting the joint from destruction [6]. Although NOR affects the musculoskeletal system in multiple ways, to our knowledge, there is no research on how NOR regulates the bone fracture healing process. In this study, we generated a tibia fracture mouse model to examine the role of NOR on bone regeneration as well as the mechanisms under it. For in vitro study, BMSCs from mice were used to reveal that NOR inhibits chondrocyte differentiation. 

## 2. Materials and Methods

### 2.1. Mice

Wild-type Male C57BL6/J mice were obtained from Spfbiotech (Beijing, China) and housed under specific pathogen-free conditions in an environmentally controlled clean room (temperature: 23 ± 1 °C, humidity: 50 ± 10%), with a 12 h/12 h light/dark cycle. 

### 2.2. Tibia Fracture Model

For tibia fractures, 8-week-old mice were anesthetized with ketamine (35 mg/kg, Covetrus North America, Portland, ME, USA) and xylazine (4.5 mg/kg, Akom, Lake Forest, IL, USA). The surgery was performed on the right leg, with the skin under the knee sterilized with 70% ethanol, and then a 6–7 mm long incision of the skin was made. A 27 G sterile needle was used to make an entry point through the proximal tibial articular surface, and then a tibial midshaft transection was made using a circular saw before the 27 G needle was reinserted into the medullary canal of the tibia. To reattach the fractured tibia, the wound was closed with suture and mice were housed separately for recovery. Norisoboldine (Absin, Shanghai, China) extracted from *Lindera aggregata* with a purity ≥98% (HPLC) was injected intraperitoneally (IP) daily starting from the next day post-surgery at a dose of 20 mg/kg [14], while the control group were injected with DMSO (Solarbio, Beijing, China), and the mice were sacrificed 7 and 14 days after surgery.

### 2.3. BMSC Cell Cultures

BMSCs were isolated from 8- to 12-week-old WT mice, as previously reported [15]. Briefly, the bone marrow of the femur and tibia was flushed out with PBS and incubated in a basic medium [α-MEM (Corning, NY, USA) containing 10% FBS (Corning, USA) and 1% penicillin/streptomycin]. The cells were incubated at 37 °C in a 5% (*v*/*v*) CO_2_ incubator, and the medium was changed every 2 days.

### 2.4. Chondrogenic Induction of BMSCs In Vitro

P2 BMSCs with good growth conditions were cultured in 12-well plates at a density of 5 × 10^5^. After the BMSCs reached 80% confluence, they were incubated from basic medium to chondrogenic differentiation medium [high-glucose DMEM (Corning, USA) with the addition of 10% FBS, 1% P/S, 40 mg/mL proline (Solarbio, Beijing, China), 100 mg/mL sodium pyruvate (Solarbio, China), 50 mg/mL vitamin C (Sigma Aldrich, St. Louis, MO, USA), 50 mg/mL insulin–transferrin–selenium (ThermoFisher Scientific, Waltham, MA, USA), and 10 ng/mL transforming growth factor (Abbkine, Wuhan, China)] [16]. The chondrogenic induction medium was changed every 2 days for 7 and 14 days.

### 2.5. Micro-Computed Tomography (µCT) Analysis

Mice were sacrificed and the tibia were isolated and fixed in 4% paraformaldehyde (PFA, Solarbio, China) buffer for 24 h before storing in 70% ethanol. High-resolution μCT scanning (SkyScan1172, Bruker-μCT, Kontich, Belgium) under 65 kV and 153 μA with a 10 μm resolution and 1.0 mm aluminum filter was used to scan the tibia for analysis. NRecon v1.6 software (Bruker) and CTAn v1.9 (Bruker) were used for image reconstruction and quantitative analysis, respectively. Regions of interest (ROI) were determined by manually selecting trabecular bone and callus areas.

### 2.6. Histology

For histological analysis, the fractured tibia was decalcified, paraffin was embedded, and the tibia was cut into 10 μm sections. Paraffin sections were rehydrated and stained with hematoxylin (Servicebio, Wuhan, China) for 1 min. After rinsing with water, the sections were stained with 0.2% Fast Green solution (Sigma Aldrich, USA), then incubated in 1% acetic acid for 12 s. Next, the sections were stained with 0.1% Safranin O solution (Sigma Aldrich, USA) for 3 min. The slides were washed, dehydrated, and mounted with resin mounting solution before taking images. 

### 2.7. Wound Healing Assay

BMSCs were cultured in 12-well culture plates. Cells were grown until the cell fusion rate was 100%, and then a straight line was drawn vertically in the center of the well with a sterile 200 μm pipette tip. The decidual cells were removed by rinsing with PBS and added to normal medium and drug-containing medium, respectively. Cell migration was recorded at 0, 3, 6, 9, 12, and 24 h. Cells were photographed under an inverted fluorescent microscope (Leica, Weztlar, Germany).

### 2.8. Alcian Blue Staining

For Alcian Blue staining, monolayers of cultured cells were rinsed with PBS and fixed with 4% PFA (Solarbio, China) solution for 15 min. After fixation, the cells were rinsed with PBS and stained with 1% Alcian Blue (Sigma Aldrich, USA) in 0.1 N hydrochloric acid (HCl) for 30 min. Then, the cells were rinsed three times with 0.1 N HCl. After neutralizing the acidity with distilled water, sample images were taken under inverted fluorescence microscopy (Leica, Germany).

### 2.9. Immunofluorescent (IF) Staining

For IF staining, antigen retrieval was conducted on paraffin sections in citrate antigen retrieval solution (Sangon Biotech, Shanghai, China) for 10 min at 56 °C then transferred to 99 °C for 3 min. After cooling down for 30 min at room temperature (RT), sections were treated with 0.1% Triton X-100 (Solarbio, China) for 5 min, then incubated with block solution [3% BSA (Sigma Aldrich, USA), 3% goat serum (Solarbio, China), plus 0.01% TritonX-100] for 1 h. After incubation with primary antibody overnight at 4 °C, and with fluorescent secondary antibody (Abcam, Cambridge, MA, USA) for 1 h at RT, samples were imaged with a confocal fluorescence microscope (Carl Zeiss, Oberkochen, Germany). Primary antibody dilution was 1:400 for OSX (SC-393060, Santa Cruz, CA, USA) and 1:500 for COL1 (GB11022-3, Servicebio, Wuhan, China).

### 2.10. RNA Extraction and Quantitative PCR

Total RNA extraction was performed with TRIzol reagent (ThermoFisher Scientific, USA) and reverse transcribed into cDNA using PrimeScript RT kit (TaKaRa, Kusatsu, Shiga, Japan). Real-time PCR (RT-PCR) was performed on an Applied Biosystems real-time fluorescence quantitative PCR instrument. The primer sequences used in this study are shown in Table 1.

### 2.11. Statistical Analysis

Student’s *t*-test was used to compare two independent groups. Statistical analyses were performed using GraphPad Prism 8 data and expressed as mean ± SEM. Differences were considered statistically significant if *p* < 0.05.

## 3. Results

### 3.1. NOR Inhibits Bone Regeneration by Delaying Callus Formation

**Figure 1:**
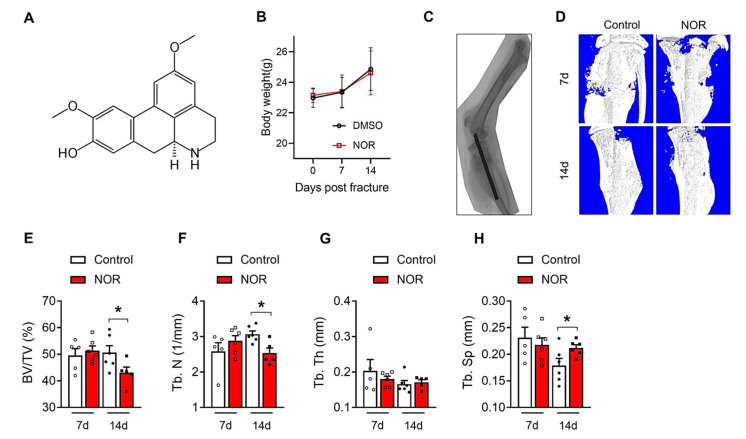
NOR treatment inhibited callus formation in mice tibia fracture healing; (**A**) chemical structure of NOR; (**B**) body weight change at 7 and 14 days post-fracture; (**C**) schematic of a mouse tibial fracture following intramedullary pinning; (**D**) representative computer renderings of bone structure of the fracture sites at days 7 and 14 post-fracture; (**E**–**H**) quantitative analysis of callus after micro-CT scanning (male, *n* = 5–6): (**E**) BV/TV (%); (**F**) Tb. N (1/mm); (**G**) trabecular thickness (Tb. Th) (mm); (**H**) Tb. Sp (mm). * *p* < 0.05.

Firstly, we generated a tibia fracture model at the proximal third of the tibial diaphysis with a dental disc saw. After the fracture, because of the poor bioavailability and poor absorption rate of NOR [11], we performed the IP injection of NOR every day. The chemical structure of NOR is shown in Figure 1A, according to the PubChem database. The mice were sacrificed 7 and 14 days post-surgery, and the callus formation was measured. Body weight was observed at each time point and was found to be not influenced by NOR treatment. (Figure 1B). As shown in Figure 1C–G, micro-CT analysis of the tibia fracture site revealed that callus mineralization was similar 7 days after fracture. However, mineralized callus formation was significantly decreased in the NOR-treated group 14 days after fracture, indicated by decreased bone volume/tissue volume (BV/TV) (Figure 1D), trabecular number (Tb. N) (Figure 1E), and increased trabecular separation (Tb. Sp) (Figure 1G). A mineralized callus bridged the fracture gap, and a reduced callus size in a fractured bone may be caused by the retarded bone remodeling process. Therefore, these data suggested that NOR is a negative regulator of fracture healing and delays endochondral ossification.

### 3.2. NOR Decreases Cartilage Formation by Inhibiting Differentiation of BMSC to Chondrocyte

**Figure 2:**
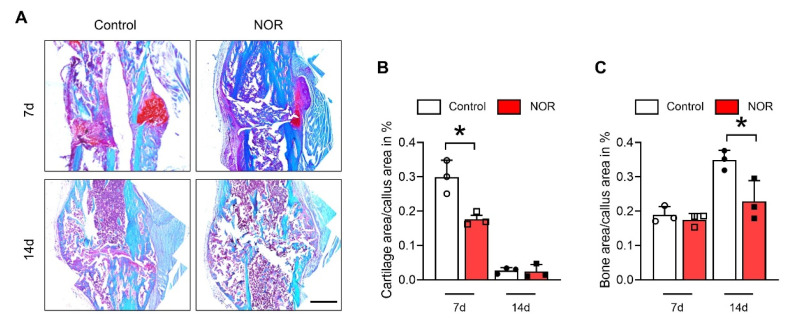
NOR suppresses cartilage formation and chondrogenic differentiation of BMSCs. (**A**) Safranin O staining of the fracture site at 7 and 14 days post-fracture. (Scale bar, 500 μm). (**B**) Relative cartilage area in the fracture callus at 7, 14, and 21 days after fracture. (**C**) Relative bone mineral area in the fracture callus at 7 and 14 days after fracture. * *p* < 0.05.

Safranin O staining of the histology sections at the fractures area confirmed a decrease in cartilage formation in the NOR-treated group (Figure 2A). It was shown that the cartilage formation area was smaller in the NOR group on day 7 (Figure 2B), then the cartilage area decreased in both groups as the mineral area increased because of the ossification process at day 14 (Figure 2B,C). The results indicating NOR have a negative effect on chondrogenesis, which might be caused by the inhibition of the bone marrow mesenchymal stem cells (BMSCs) differentiation into chondrocytes.

**Figure 3:**
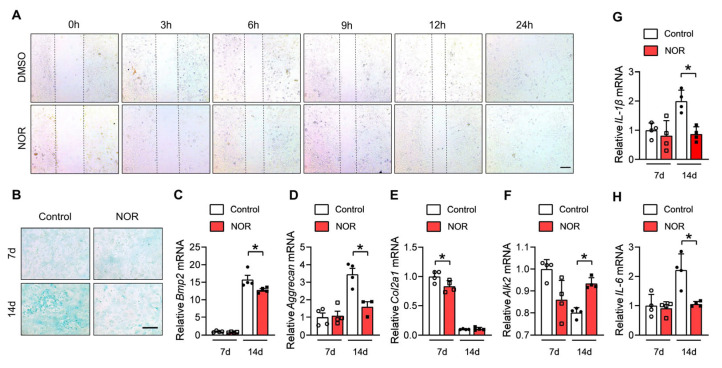
NOR suppresses the ability of BMSCs differentiation into chondrocytes. (**A**) Wound healing assay representing cell migration ability in control and NOR group. (Scale bar, 100 μm). (**B**) Alcian blue staining of the BMSCs incubated in chondrogenic medium for 14 days. (Scale bar, 200 μm). (**C**–**H**) *Bmp2*, *Aggrecan*, *Col2a1*, *Alk2*, *IL-1β* and *IL-6* mRNA levels in 7 and 14 days differentiated BMSCs. * *p* < 0.05.

To confirm this hypothesis, we conducted a wound healing assay in vitro. The migration ability of NOR-treated BMSCs did not change at all the timepoints compared with the control group (Figure 3A). Next, we measured the chondrogenic effect of NOR on BMSCs. Primary BMSCs were treated for 14 days with 10 μM NOR or DMSO in chondrogenic medium, and less proteoglycans in the NOR group were detected by Alcian Blue staining (Figure 3B). The genes involved in chondrogenesis were analyzed on day 0 and 14 after NOR treatment using RT-PCR. We detected a level of bone morphogenetic protein 2 (BMP2) (Figure 3C), which is a growth factor for the chondrogenic induction of MSCs [17], and a chondrocyte phenotype marker named Aggrecan (Figure 3D) and type 2 collagen (Col2a1) (Figure 3E) [18]. Consistent with the alcian blue staining result, the expression of *Bmp2, Aggrecan*, and *Col2a1* was significantly downregulated in NOR-incubated cells on day 7 or day 14. To prove that the change in *Bmp2* is biologically significant, we tested its downstream factors Alk2 (Figure 3F), which is a BMP type I receptor, and found that it was increased on day 14 because of the negative feedback loops of molecular interaction.

The anti-inflammatory effect of NOR was also detected in vitro. RT-PCR revealed that *IL-1β* (Figure 3G) and *IL-6* (Figure 3H) mRNA were both inhibited by NOR after chondrogenic differentiation for 14 days.

### 3.3. NOR Decreases Bone Ossification by Inhibiting Osterix (OSX)–Collage I (COL1) Signaling

**Figure 4:**
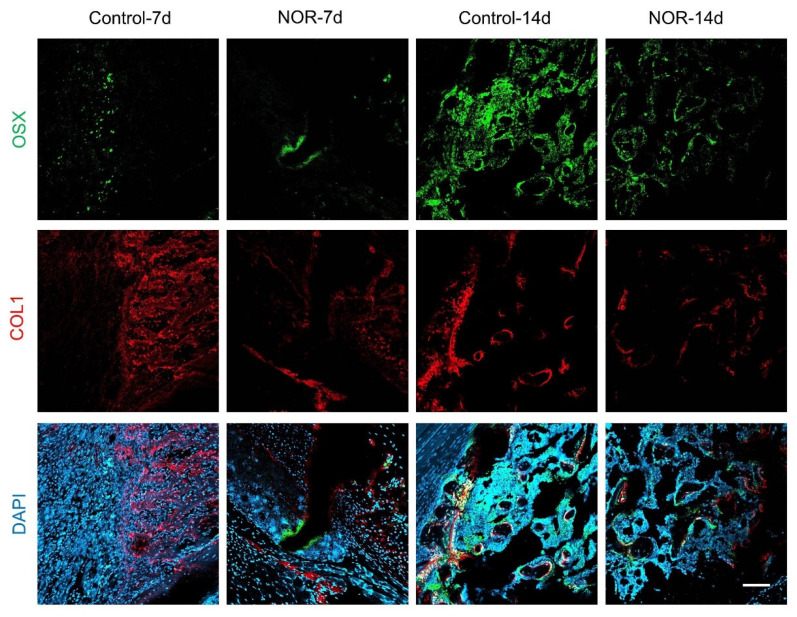
NOR suppressed OSX and COL1 signaling at the fracture site. Tibias from fracture model were co-stained for OSX (**green**) and COL1 (**red**) at 7 or 14 days after surgery. (Scale bar: 100 μm).

Less cartilage formation in fracture healing suggests less endochondral ossification. To examine the molecular signaling changes, immunofluorescent staining was performed at the callus. The pre-osteoblast marker (OSX) and functional osteoblast marker COL1 [19] were detected at the fracture site. Consistent with less bone remodeling in NOR-treated mice, OSX and COL1 were both decreased in the NOR group (Figure 4). Herein, as a natural product of Chinese herbal medicine, NOR decreases the bone fracture healing process via diminishing cartilage formation and endochondral ossification.

## 4. Discussion

Fracture healing predominantly occurs through the process of endochondral ossification [20], which involves the formation of cartilage and the transition of cartilage to bone [21]. Therefore, chondrogenesis, which directly affects cartilage formation, is an important component for fracture repair. In some physiological status like diabetes [22,23] or the disruption of glucocorticoid signaling [24], fracture healing is impaired by inhibited cartilage development.

Except for producing cartilage, chondrocytes produce factors like FOXO1 to modulate vessel formation and fracture healing [25]. BMSCs act as a relevant cell source for the regeneration of focal cartilage and bone lesions since they are multipotent cells with the capacity to differentiate into osteoblasts, chondrocytes, fibroblasts, myocytes, and adipocytes [26]. The chondrocytes at the fracture site are derived from MSCs migrated from the periosteum and bone marrow. The recruitment of MSCs in the fracture repair is under molecular regulation by cytokines released at the fracture site, particularly CXCL12, also known as stromal-cell-derived factor 1 (SDF1). SDF1 increased callus formation as well as the induced expression of VEGF and Runx2 in the soft tissue callus [27,28]. Notch signaling is another potentially important factor in both regulating MSC number and activation [29]. For the cartilage regeneration, BMP2 is one of the main chondrogenic growth factors which induces chondrogenic differentiation and osteogenic differentiation in MSCs [30,31]. To our knowledge, this research is the first time to demonstrate that NOR could inhibit BMP2-Col2a1/Aggrecan signaling in BMSCs to suppress the differentiation of MSCs to chondrocytes in the fracture repair. 

NOR has an anti-inflammatory effect on arthritis, so NOR may have a different effect on aged animals because of heavier inflammation with aging. The traditional Chinese medicine NOR for bone remodeling in different sexes and ages still requires more investigation in the future. A negative effect on fracture healing should be considered when doctors plan to use this herbal medicine in osteoporosis or arthritis patients. 

As a major constituent of Chinese herbal medicine Radix Linderae, the function of NOR is investigated in many kinds of diseases, such as acute lung injury, colitis, rheumatoid arthritis, etc. [7,13,32]. Although NOR has an anti-inflammatory effect on arthritis, it suppresses the fracture healing effect on a young mouse model by inhibiting chondrogenesis. In summary, this research shows that NOR inhibits mice fracture healing via inhibiting the differentiation of BMSCs to chondrocytes at the fracture site, and the downstream effects of NOR are mediated via BMP2-Col2a1/Aggrecan signaling during differentiation. Moreover, the osteogenic signal is weaker because of less callus formation under NOR treatment. Taken together, these results demonstrate the novel inhibitory effect of NOR in the transition of BMSCs to chondrocytes, thereby providing a new understanding of how to use this traditional medicine under different body conditions. 

## 5. Conclusions

NOR inhibits mice fracture healing by suppressing the differentiation of BMSCs to chondrocytes via BMP2 signaling. These findings imply that NOR intake might delay the union of the fractures and should be noticed when used in other diseases.

## Figures and Tables

**Table 1:** Oligonucleotides used in RT-PCR.

Gene	Forward	Reverse
*Aggrecan*	CCTGCTACTTCATCGACCCC	AGATGCTGTTGACTCGAACCT
*Bmp2*	GGGACCCGCTGTCTTCTAGT	TCAACTCAAATTCGCTGAGGAC
*Gapdh*	AGGTCGGTGTGAACGGATTTG	TGTAGACCATGTAGTTGAGGTCA
*Col2a1*	GGGAATGTCCTCTGCGATGAC	GAAGGGGATCTCGGGGTTG
*Alk2*	GTGGAAGATTACAAGCCACCA	GGGTCTGAGAACCATCTGTTAGG
*Il-6*	TAGTCCTTCCTACCCCAATTTCC	TTGGTCCTTAGCCACTCCTTC
*Il-1* *β*	GCAACTGTTCCTGAACTCAACT	ATCTTTTGGGGTCCGTCAACT

## Data Availability

The datasets used and/or analyzed during the current study are available from the corresponding author on reasonable request.

## References

[1] Einhorn T.A., Gerstenfeld L.C. (2015). Fracture healing: Mechanisms and interventions. Nat. Rev. Rheumatol..

[2] Gerstenfeld L.C., Cho T.J., Kon T., Aizawa T., Tsay A., Fitch J., Barnes G.L., Graves D.T., Einhorn T.A. (2003). Impaired fracture healing in the absence of TNF-alpha signaling: The role of TNF-alpha in endochondral cartilage resorption. J. Bone Miner. Res. Off. J. Am. Soc. Bone Miner. Res..

[3] Wang J., Wei Y., Zhou Z., Yang J., Jia Y., Wu H., Dong H., Leng X. (2022). Deer antler extract promotes tibia fracture healing in mice by activating BMP-2/SMAD4 signaling pathway. J. Orthop. Surg. Res..

[4] Gerstenfeld L.C., Cullinane D.M., Barnes G.L., Graves D.T., Einhorn T.A. (2003). Fracture healing as a post-natal developmental process: Molecular, spatial, and temporal aspects of its regulation. J. Cell. Biochem..

[5] Wei Z.F., Lv Q., Xia Y., Yue M.F., Shi C., Xia Y.F., Chou G.X., Wang Z.T., Dai Y. (2015). Norisoboldine, an Anti-Arthritis Alkaloid Isolated from Radix Linderae, Attenuates Osteoclast Differentiation and Inflammatory Bone Erosion in an Aryl Hydrocarbon Receptor-Dependent Manner. Int. J. Biol. Sci..

[6] Luo Y., Liu M., Xia Y., Dai Y., Chou G., Wang Z. (2010). Therapeutic effect of norisoboldine, an alkaloid isolated from Radix Linderae, on collagen-induced arthritis in mice. Phytomed. Int. J. Phytother. Phytopharm..

[7] Chen Q., Shao X., He Y., Lu E., Zhu L., Tang W. (2021). Norisoboldine Attenuates Sepsis-Induced Acute Lung Injury by Modulating Macrophage Polarization via PKM2/HIF-1alpha/PGC-1alpha Pathway. Biol. Pharm. Bull..

[8] Gao S., Li W., Lin G., Liu G., Deng W., Zhai C., Bian C., He G., Hu Z. (2016). Norisoboldine, an alkaloid from Radix linderae, inhibits NFAT activation and attenuates 2,4-dinitrofluorobenzene-induced dermatitis in mice. Immunopharmacol. Immunotoxicol..

[9] Lu Q., Tong B., Luo Y., Sha L., Chou G., Wang Z., Xia Y., Dai Y. (2013). Norisoboldine suppresses VEGF-induced endothelial cell migration via the cAMP-PKA-NF-kappaB/Notch1 pathway. PLoS ONE.

[10] Wei Z.F., Tong B., Xia Y.F., Lu Q., Chou G.X., Wang Z.T., Dai Y. (2013). Norisoboldine suppresses osteoclast differentiation through preventing the accumulation of TRAF6-TAK1 complexes and activation of MAPKs/NF-kappaB/c-Fos/NFATc1 Pathways. PLoS ONE.

[11] Tong B., Dou Y., Wang T., Yu J., Wu X., Lu Q., Chou G., Wang Z., Kong L., Dai Y. (2015). Norisoboldine ameliorates collagen-induced arthritis through regulating the balance between Th17 and regulatory T cells in gut-associated lymphoid tissues. Toxicol. Appl. Pharmacol..

[12] Wei Z., Wang F., Song J., Lu Q., Zhao P., Xia Y., Chou G., Wang Z., Dai Y. (2012). Norisoboldine inhibits the production of interleukin-6 in fibroblast-like synoviocytes from adjuvant arthritis rats through PKC/MAPK/NF-kappaB-p65/CREB pathways. J. Cell. Biochem..

[13] Lu Q., Lu S., Gao X., Luo Y., Tong B., Wei Z., Lu T., Xia Y., Chou G., Wang Z. (2012). Norisoboldine, an alkaloid compound isolated from Radix Linderae, inhibits synovial angiogenesis in adjuvant-induced arthritis rats by moderating Notch1 pathway-related endothelial tip cell phenotype. Exp. Biol. Med..

[14] Xing D., Li Q., Lin G., Lin H., Kang W., Zhang M., Ding R., Li N. (2022). The protective effects of propofol against renal ischemia-reperfusion injury are potentiated by norisoboldine treatment via inhibition of oxidative stress pathways. J. Biochem. Mol. Toxicol..

[15] Song J.H., Kim J.W., Lee M.N., Oh S.H., Piao X., Wang Z., Kwon S.H., Kim O.S., Koh J.T. (2022). Isolation of High Purity Mouse Mesenchymal Stem Cells through Depleting Macrophages Using Liposomal Clodronate. Tissue Eng. Regen. Med..

[16] Shao R., Zhang Z., Xu Z., Ouyang H., Wang L., Greenblatt M., Chen X., Zou W. (2021). H3K36 methyltransferase NSD1 regulates chondrocyte differentiation for skeletal development and fracture repair. Bone Res..

[17] Zhao C., Jiang W., Zhou N., Liao J., Yang M., Hu N., Liang X., Xu W., Chen H., Liu W. (2017). Sox9 augments BMP2-induced chondrogenic differentiation by downregulating Smad7 in mesenchymal stem cells (MSCs). Genes Dis..

[18] Legendre F., Ollitrault D., Gomez-Leduc T., Bouyoucef M., Hervieu M., Gruchy N., Mallein-Gerin F., Leclercq S., Demoor M., Galera P. (2017). Enhanced chondrogenesis of bone marrow-derived stem cells by using a combinatory cell therapy strategy with BMP-2/TGF-beta1, hypoxia, and COL1A1/HtrA1 siRNAs. Sci. Rep..

[19] Nakashima K., Zhou X., Kunkel G., Zhang Z., Deng J.M., Behringer R.R., de Crombrugghe B. (2002). The novel zinc finger-containing transcription factor osterix is required for osteoblast differentiation and bone formation. Cell.

[20] Hu D.P., Ferro F., Yang F., Taylor A.J., Chang W., Miclau T., Marcucio R.S., Bahney C.S. (2017). Cartilage to bone transformation during fracture healing is coordinated by the invading vasculature and induction of the core pluripotency genes. Development.

[21] Kodama J., Wilkinson K.J., Iwamoto M., Otsuru S., Enomoto-Iwamoto M. (2022). The role of hypertrophic chondrocytes in regulation of the cartilage-to-bone transition in fracture healing. Bone Rep..

[22] Ogasawara A., Nakajima A., Nakajima F., Goto K., Yamazaki M. (2008). Molecular basis for affected cartilage formation and bone union in fracture healing of the streptozotocin-induced diabetic rat. Bone.

[23] Kayal R.A., Alblowi J., McKenzie E., Krothapalli N., Silkman L., Gerstenfeld L., Einhorn T.A., Graves D.T. (2009). Diabetes causes the accelerated loss of cartilage during fracture repair which is reversed by insulin treatment. Bone.

[24] Tu J., Henneicke H., Zhang Y., Stoner S., Cheng T.L., Schindeler A., Chen D., Tuckermann J., Cooper M.S., Seibel M.J. (2014). Disruption of glucocorticoid signaling in chondrocytes delays metaphyseal fracture healing but does not affect normal cartilage and bone development. Bone.

[25] Zhang C., Feinberg D., Alharbi M., Ding Z., Lu C., O’Connor J.P., Graves D.T. (2019). Chondrocytes Promote Vascularization in Fracture Healing Through a FOXO1-Dependent Mechanism. J. Bone Miner. Res. Off. J. Am. Soc. Bone Miner. Res..

[26] Bahney C.S., Zondervan R.L., Allison P., Theologis A., Ashley J.W., Ahn J., Miclau T., Marcucio R.S., Hankenson K.D. (2019). Cellular biology of fracture healing. J. Orthop. Res. Off. Publ. Orthop. Res. Soc..

[27] Kitaori T., Ito H., Schwarz E.M., Tsutsumi R., Yoshitomi H., Oishi S., Nakano M., Fujii N., Nagasawa T., Nakamura T. (2009). Stromal cell-derived factor 1/CXCR4 signaling is critical for the recruitment of mesenchymal stem cells to the fracture site during skeletal repair in a mouse model. Arthritis Rheum..

[28] Li X., Gao Z., Wang J. (2011). Single percutaneous injection of stromal cell-derived factor-1 induces bone repair in mouse closed tibial fracture model. Orthopedics.

[29] Wang C., Inzana J.A., Mirando A.J., Ren Y., Liu Z., Shen J., O’Keefe R.J., Awad H.A., Hilton M.J. (2016). NOTCH signaling in skeletal progenitors is critical for fracture repair. J. Clin. Investig..

[30] Shu B., Zhang M., Xie R., Wang M., Jin H., Hou W., Tang D., Harris S.E., Mishina Y., O’Keefe R.J. (2011). BMP2, but not BMP4, is crucial for chondrocyte proliferation and maturation during endochondral bone development. J. Cell Sci..

[31] Zhou N., Li Q., Lin X., Hu N., Liao J.Y., Lin L.B., Zhao C., Hu Z.M., Liang X., Xu W. (2016). BMP2 induces chondrogenic differentiation, osteogenic differentiation and endochondral ossification in stem cells. Cell Tissue Res..

[32] Lv Q., Wang K., Qiao S.M., Dai Y., Wei Z.F. (2018). Norisoboldine, a natural aryl hydrocarbon receptor agonist, alleviates TNBS-induced colitis in mice, by inhibiting the activation of NLRP3 inflammasome. Chin. J. Nat. Med..

